# Adherence to the integrated management of childhood illness guidelines in Namibia, Kenya, Tanzania and Uganda: evidence from the national service provision assessment surveys

**DOI:** 10.1186/s12913-017-2781-3

**Published:** 2017-12-13

**Authors:** Carsten Krüger, Monika Heinzel-Gutenbrunner, Mohammed Ali

**Affiliations:** 1 Department of Paediatrics, Witten/Herdecke University, Witten, Germany; 2 Children‘s Hospital, St. Franziskus Hospital, Robert-Koch-Strasse 55, D-59227 Ahlen, Germany; 3 MH Statistical Consulting, Marburg, Germany; 4 Faculty of Health Sciences, School of Nursing, Midwifery & Paramedicine, Curtin University, Perth, Australia

**Keywords:** Integrated management of childhood illness, Adherence, Health workers, Sub-Saharan Africa

## Abstract

**Background:**

Integrated Management of Childhood Illness (IMCI) is regarded as a standard public health approach to lowering child mortality in developing countries. However, little is known about how health workers adhere to the guidelines at the national level in sub-Saharan African countries.

**Methods:**

Data from the Service Provision Assessment surveys of Namibia (NA) (survey year: 2009), Kenya (KE) (2010), Tanzania (TZ) (2006) and Uganda (UG) (2007) were analysed for adherence to the IMCI guidelines by health workers. Potential influencing factors included the survey country, patient’s age, the different levels of the national health system, the training level of the health care provider (physician, non-physician clinician, nurse-midwife, auxiliary staff), and the status of re-training in IMCI.

**Results:**

In total, 6856 children (NA: 1495; KE: 1890; TZ: 2469; UG: 1002 / male 51.2–53.5%) aged 2–73 months (2–24 months, 65.3%; median NA: 19 months; KE: 18 months; TZ: 16 months; UG: 15 months) were clinically assessed by 2006 health workers during the surveys.

Less than 33% of the workers carried out assessment of all three IMCI danger signs, namely inability to eat/drink, vomiting everything, and febrile convulsions (NA: 11%; KE: 11%; TZ: 14%; UG: 31%) while the rate for assessing all three of the IMCI main symptoms of cough/difficult breathing, diarrhoea, and fever was < 60% (NA: 48%; KE: 34%; TZ: 50%; UG: 57%). Physical examination rates for fever (temperature) (NA: 97%; KE: 87%; TZ: 73%; UG: 90%), pneumonia (respiration rate/auscultation) (NA: 43%; KE: 24%; TZ: 25%; UG: 20%) and diarrhoea (dehydration status) (NA: 29%; KE: 19%; TZ: 20%; UG: 39%) varied widely and were highest when assessing children with the actual diagnosis of pneumonia and diarrhoea.

Adherence rates tended to be higher in children ≤ 24 months, at hospitals, among higher-qualified staff (physician/non-physician clinician) and among those with recent IMCI re-training.

**Conclusion:**

Despite nationwide training in IMCI the adherence rates for assessment and physical examination remained low in all four countries. IMCI training should continue to be provided to all health staff, particularly nurses, midwives, and auxiliary staff, with periodic re-training and an emphasis to equally target children of all age groups.

## Background

Despite considerable progress towards Millennium Development Goal IV, children under five still die in large numbers [1]. The Global Burden of Disease study estimated that in 2015 5.8 million deaths occurred in this age group [2]. Improving quality of care in child health services will be essential for further substantial reductions in under-five child mortality, now included in Sustainable Development Goal III [3–5]. In this regard, outpatient care is particularly important, as it often constitutes the first point of contact between the sick child and the health system [4–7]. Some 10–30% of these children may need hospital admission during the course of their illness, thus good service provision is essential in the outpatient setting [5, 7, 8].

In the 1990s, WHO and UNICEF developed the Integrated Management of Childhood Illness (IMCI) strategy to improve quality of child health care and reduce under-five mortality [5–7]. It has now been introduced into the health systems of over 100 countries [5, 9]. Initial evaluations, conducted in IMCI study areas in Bangladesh, Brazil, South Africa, Tanzania and Uganda, demonstrated improvement in the provision of child care [10–17]. Later on several studies at the local, district and regional level in sub-Saharan Africa, Asia and South America provided mixed results concerning the effects on child health and health workers’ performance [17–30]. One major concern emerging from these studies was that health workers often would not adhere to the IMCI guidelines [21–24, 27, 31–35].

Adherence to guidelines and algorithms is a complex process. It is influenced by contextual factors that may affect the health workers’ extrinsic and intrinsic capacity and/or motivation to adhere to these guidelines [35]. Up till now, little is known about adherence to these guidelines at the national level in sub-Saharan African countries, despite these countries being among the first that introduced IMCI into their national health systems [5, 7]. Therefore, we analysed the situation in Namibia, Kenya, Tanzania and Uganda which adopted the IMCI policy between 1996 and 2000. For the purpose of this study, we analysed representative data from the national Service Provision Assessment (SPA) surveys conducted between 2006 and 2010, on average 10 years after the introduction of IMCI into the respective country [36–39].

## Methods

### Selection of IMCI countries

We selected the four Low-and-Middle-Income (LMIC) countries from sub-Saharan Africa because their IMCI programmes were already well established at the time of the national SPA survey and the survey data were accessible for secondary analysis. Kenya, Tanzania and Uganda were quite comparable with regards to their health systems and population structure, whereas Namibia fared much better than these three countries with regards to skilled attendance at birth, mortality rates, numbers of health workers and health expenditure (Table 1) [36–43]. At the time of the SPA surveys IMCI was used in between 60 to 100% of all districts in these countries (Table 1).

**Table 1:** Background information on the survey countries (corresponding to survey year) [36–43]

	Namibia	Kenya	Tanzania	Uganda
Human development index	120	143	159	154
Country size (km^2^)	824,116	580,367	945,087	241,040
Population (million)	2.2	40.9	38.5	28.8
Children < 5 years (million)	0.279	6.664	6.953	6.028
Annual births (×1000)	59	1530	1408	1468
Skilled birth attendance (%)	81	42	43	39
Neonatal mortality rate (/1000)	19	27	43	32
Under-five mortality rate (/1000)	48	85	118	130
Maternal mortality ratio (/100,000)	180	360	790	430
Physicians per population (/10,000)	3.39	1.7	0.73	1.04
Total number of health workers	6600	80,464	47,000	27,487
Health expenditure/capita (USD)	467	72	29	135
Number of hospitals/health centres/dispensaries	46/49/351	450/860/4882	219/481/4679	119/164/2717
Start of IMCI (year)	1999	2000	1996	1996
IMCI coverage in districts (%)	60 (20/34)	75 (56/75)	100 (all)	100 (all)
SPA survey (year)	2009	2010	2006	2007
Number of hospitals/health centres/dispensaries in survey	45/47/319	253/153/297	128/41/442	119/81/291

*IMCI* Integrated management of childhood illness, *SPA* Service provision assessment, *USD* US dollars

### Data collection

Macro International (www.dhsprogram.com), in partnership with the respective ministries of health, conducted the SPA surveys. They employed similar data collection tools across the countries [36–39]. SPA surveys are facility-based and cross-sectional. They collect information about the service provision of facilities associated with maternal, neonatal, child, and reproductive health services as well as certain infectious diseases programs (sexually transmitted infections, HIV/AIDS, tuberculosis, malaria).

The sampling process has been described in detail elsewhere [36–39]. In short, the ministry of health provided a list of all health facilities in the country. A representative sample (~ 10–15%) was selected from this list, including all hospitals down to the district hospital level, which resulted in an oversampling of these institutions. The remaining institutions (health centres, dispensaries) were sampled in such a way as to represent different providers and regions of each country. In Namibia all 446 health facilities were included on purpose, although only 411 could be surveyed (92%) (Table 1).

At the selected facilities, only health care providers were included who were present on the day of the survey and had examined and/or provided care to the patients. A maximum of eight providers were selected per institution. The selection of children (and their caretakers) was opportunistic, meaning that they were selected as they came in without any recruitment screening or randomisation. As per study protocol, five patients were to be sampled per provider.

### Data selection

As dependent variables, we analysed rates of directly observed adherence to IMCI guidelines by health staff focussing on the main diagnostic components in the IMCI algorithms (treatment and other items will be analysed in a separate paper) [44]. These components were (1) the three IMCI danger signs of ‘inability to eat/drink’, ‘vomiting everything’, and ‘history of febrile convulsions’ as single items and in combination (data on being lethargic, unconscious or convulsing at presentation were not recorded); (2) the three IMCI main symptoms of ‘cough/difficult breathing’, ‘diarrhoea’, and ‘fever’ as single items and in combination; and (3) basic elements of physical examination (measuring temperature and/or feeling for fever, counting respiration rate and/or chest auscultation, and assessing dehydration), all among children aged 2 months or older. Infants younger than 2 months were excluded from the analyses as they were not covered by the main IMCI system, but rather by a separate neonatal IMCI tool which was not part of the surveys [36–39, 44]. In addition, the time the health staff spent on covering all parts of the IMCI algorithm (assessment, classification, treatment, vaccination, nutrition counselling, caregiver instruction) was retrieved from the data base. The survey observers did not play any part in the consultation process and did not assess whether the diagnosis or treatment was appropriate for the patient.

Independent variables for comparison included the survey country, patient’s age (younger vs. older than 24 months), the different levels of the national health system (hospital, health centre, dispensary), and the training level of the health care provider (physician, non-physician clinician (NPC) (defined as those who have fewer clinical skills than physicians but more than nurses/midwives) [45], nurse-midwife, auxiliary staff (e.g. auxiliary nurse, medical attendant, pharmacist, pharmaceutical technician/assistant, laboratory technologist/technician/assistant, nutritionist/nutrition technician, health education officer)). We further divided the health workers into three different groups according to recent (within the last 12 months), more distant (preceding 13–36 months) or no re-training in IMCI.

To achieve comparability across the different countries, we re-grouped the three levels of the health system in Namibia, Kenya and Uganda into hospital, health centre, and dispensary categories. Health care providers were also grouped into the same categories (in all countries). Details of this process are available on request.

### Statistical analysis

Frequencies and rates (median, interquartile range (IQR)) were computed for the respective categories at country, child’s age, health system, provider, and IMCI re-training levels. We used IBM^®^ SPSS^®^ Statistics version 24 for all analyses.

### Ethical clearance

Macro International as the copyright holder granted the authors unrestricted access to all survey data, which were completely anonymised, for this secondary analysis after submission of the research proposal. For such an analysis, no approval of an ethical committee is required according to the Good Practice of Secondary Data Analysis, a national guideline for the use of epidemiologic databases in Germany, which was fully adhered to [46].

The surveys themselves had been initiated, planned and approved by the respective Ministry of Health. During the actual surveys, parents and health staff had given informed written consent to direct observation of the assessment process [36–39].

## Results

In the four surveys, 7304 sick child assessments (Namibia: 1578; Kenya: 2049; Tanzania: 2565; Uganda: 1578) were observed; these also included children younger than 2 months. As mentioned earlier, those younger than 2 months were excluded from the analysis. Thus 6856 children with a slight male preponderance, aged 2–73 months (2–24 months: 65.3%), were assessed during the surveys (Table 2).

**Table 2:** Demographic characteristics of survey participants

	Namibia (*n* = 1495)	Kenya (*n* = 1890)	Tanzania (*n* = 2469)	Uganda (*n* = 1002)
Sex				
- Male (%)	53.5	52.3	52.1	51.2
- Female (%)	46.5	47.7	47.9	48.8
Age (months)				
- Median	19	18	16	15
- Interquartile range	10–33	10–32	9–30	9–28
- 2-24 months (%)	60.9	64.7	67.2	68.2

Across the countries, a total of 2006 health service providers of all cadres were observed during their consultations (Table 3). The vast majority of patients were seen by NPCs (Kenya, Tanzania, Uganda) and nurses/midwives (especially in Namibia). The IMCI re-training status was known in only 62% of the health workers. The re-training level varied between the countries with Namibia and Kenya performing worst, and among the different provider strata with good coverage for physicians and NPCs in Tanzania and Uganda, but considerably less coverage for nurses/midwives in these two countries (Table 3).

**Table 3:** Number of service providers (n), patient assessments per provider (n) and providers’ re-training status in IMCI (percentage)

	Namibia	Kenya	Tanzania	Uganda
Physicians - all (n)	19	40	46	18
Number of assessed patients (n) (total/median/IQR)	54/2/1–5	89/2/1–3.75	157/4/2–5	41/2/1–3
Physicians (with data on IMCI training available) (n)	9	36	12	11
IMCI training last 12 months (%)	44.5	31	33	45
IMCI training last 13–36 months (%)	11	22	59	55
No IMCI training (%)	44.5	47	8	0
Non-physician clinician – all (n)	3	315	414	183
Number of assessed patients (n) (total/median/IQR)	8/2/1–5	987/3/2–4	1697/5/3.75–5	617/3/2–5
Non-physician clinician (with data on IMCI training available) (n)	2	304	203	123
IMCI training last 12 months (%)	50	15	37	32
IMCI training last 13–36 months (%)	0	18	41	49
No IMCI training (%)	50	67	22	19
Nurse-midwife – all (n)	418	245	151	135
Number of assessed patients (n) (total/median/IQR)	1433/4/2–5	758/3/2–5	610/5/4–5	338/2/1–4
Nurse-midwife (with data on IMCI training available) (n)	174	241	52	70
IMCI training last 12 months (%)	24	14	15	30
IMCI training last 13–36 months (%)	24	19	39	29
No IMCI training (%)	52	67	46	41
Auxiliary staff^a^ - all (n)	–	15	1	3
Number of assessed patients (n) (total/median/IQR)	–	56/4/2–5	5/5/5–5	6/2/2–2
Auxiliary staff^a^ (with data on IMCI training available) (n)	–	5	0	0
IMCI training last 12 months (%)	–	0	–	–
IMCI training last 13–36 months (%)	–	0	–	–
No IMCI training (%)	–	100	–	–

*IMCI* Integrated management of childhood illness, *IQR* interquartile range

^a^this category did not exist in Namibia

Separate assessment rates for the IMCI danger sign ‘inability to eat/drink’ ranged from 34 to 66%, for the danger sign ‘vomiting everything’ from 50 to 66%, and for the danger sign ‘febrile convulsions’ from 17 to 40%. Combined assessment rates for all three IMCI danger signs were less than 33% (Table 4). Uganda yielded the best results.

**Table 4:** Adherence (percentage) of health workers to IMCI protocols during assessment of danger signs and main symptoms

IMCI item		Child’s age		Health system level			Health staff category			
Inability to eat/drink	Overall	≤ 24 months	> 24 months	Hospital	Health centre	Dispensary	Physician	NPC	Nurse-midwife	Auxiliary staff^a^
Namibia	43.1	47.3	36.7	41.4	48.5	42.2	37.0	75.0	43.2	–
Kenya	42.9	47.1	35.4	42.6	43.0	43.3	40.9	43.9	43.8	17.9
Tanzania	34.2	38.7	25.1	39.2	43.7	30.5	30.1	36.6	28.4	80.0
Uganda	66.0	69.0	59.6	69.2	65.1	55.5	77.5	72.3	53.3	66.7
Vomiting everything	Overall	≤ 24 months	> 24 months	Hospital	Health centre	Dispensary	Physician	NPC	Nurse-midwife	Auxiliary staff
Namibia	49.9	51.9	46.8	47.1	51.5	49.8	40.7	50.0	50.2	–
Kenya	52.9	53.4	52.0	55.9	51.3	49.1	55.1	55.9	50.5	28.6
Tanzania	58.6	59.8	56.2	67.4	52.4	54.9	52.6	63.4	46.7	80.0
Uganda	65.6	68.1	60.3	70.4	61.8	53.5	80.0	72.2	51.5	83.3
Febrile convulsions	Overall	≤ 24 months	> 24 months	Hospital	Health Centre	Dispensary	Physician	NPC	Nurse-midwife	Auxiliary staff
Namibia	18.1	18.2	17.9	16.1	16.1	18.6	13.0	37.5	18.2	–
Kenya	17.4	17.8	16.7	17.5	16.6	17.9	23.9	18.1	16.9	1.8
Tanzania	26.0	26.6	24.7	31.1	19.9	24.1	17.8	29.6	17.6	80.0
Uganda	39.7	41.6	35.7	46.0	34.0	25.2	45.0	47.5	24.9	50.0
All danger signs	Overall	≤ 24 months	> 24 months	Hospital	Health Centre	Dispensary	Physician	NPC	Nurse-midwife	Auxiliary staff
Namibia	10.8	12.0	8.9	12.5	9.6	11.0	7.4	25.0	10.8	–
Kenya	10.6	11.8	8.5	10.2	12.1	10.4	16.9	10.8	10.3	1.8
Tanzania	13.6	15.1	10.4	17.5	9.9	12.0	7.0	15.8	8.7	60.0
Uganda	31.3	33.2	27.3	35.9	27.2	20.9	41.8	37.4	18.9	33.3
Cough/difficult breathing	Overall	≤ 24 months	> 24 months	Hospital	Health Centre	Dispensary	Physician	NPC	Nurse-midwife	Auxiliary staff
Namibia	90.5	89.9	91.4	90.8	92.9	90.0	92.6	87.5	90.4	–
Kenya	82.0	83.1	80.0	84.1	79.9	80.0	86.5	84.6	78.5	76.8
Tanzania	80.6	81.2	79.3	84.7	74.3	79.2	79.6	83.0	74.4	40.0
Uganda	87.5	87.4	87.7	87.9	86.3	88.0	90.2	88.8	84.5	100
Diarrhoea	Overall	≤ 24 months	> 24 months	Hospital	Health Centre	Dispensary	Physician	NPC	Nurse-midwife	Auxiliary staff
Namibia	58.6	62.9	52.0	54.0	66.4	57.4	40.7	62.5	59.3	–
Kenya	45.0	47.8	39.9	66.8	55.0	61.9	47.2	48.5	41.1	32.1
Tanzania	62.9	65.8	56.9	46.9	43.7	42.8	59.0	64.4	59.7	60.0
Uganda	66.8	71.7	56.4	69.7	65.2	58.6	67.5	69.8	60.5	100
Fever	Overall	≤ 24 months	> 24 months	Hospital	Health Centre	Dispensary	Physician	NPC	Nurse-midwife	Auxiliary staff
Namibia	87.7	85.9	90.5	91.9	88.9	87.1	90.6	85.7	87.6	–
Kenya	87.4	87.0	87.5	87.5	88.1	86.8	92.1	87.3	88.4	69.6
Tanzania	90.4	90.7	89.8	91.2	86.9	90.4	81.9	91.3	90.2	80.0
Uganda	92.2	92.6	91.2	92.9	92.0	89.9	95.1	93.0	90.2	100
All main symptoms	Overall	≤ 24 months	> 24 months	Hospital	Health Centre	Dispensary	Physician	NPC	Nurse-midwife	Auxiliary staff
Namibia	47.8	49.8	44.5	48.3	55.4	46.1	38.9	50.0	48.1	–
Kenya	33.9	35.7	30.6	35.8	32.5	31.8	39.3	37.6	29.4	21.4
Tanzania	49.6	51.9	44.9	53.0	39.3	49.2	40.1	51.8	46.1	40.0
Uganda	56.7	61.1	47.3	58.4	56.0	51.3	58.5	59.6	50.3	100

*IMCI* Integrated management of childhood illness, *NPC* non-physician clinician

^a^this category did not exist in Namibia

Single assessment of the three main IMCI symptoms was better than for the danger signs. The main symptoms ‘cough/difficult breathing’ and ‘fever’ were assessed in more than 81% of patients in all countries. The assessment rate of ‘diarrhoea’ was considerably lower (< 70%). Combined assessment rates for the three IMCI main symptoms were better than for the danger signs, but still lower than 60% (Table 4). Again, Uganda achieved the best results, only surpassed by Namibia for the main symptom ‘cough/difficult breathing’.

There was wide variation in the physical examination rates for fever (temperature/feeling for fever), pneumonia (respiration rate/auscultation) and diarrhoea (dehydration), as can be seen in Table 5. These rates were highest for disease-specific elements of physical examination, such as respiration rate/auscultation for pneumonia and dehydration status for diarrhoea (Table 6).

**Table 5:** Adherence (percentage) of health workers to IMCI protocols during physical examination

IMCI item		Child’s age		Health system level			Health staff category			
Fever	Overall	≤ 24 months	> 24 months	Hospital	Health Centre	Dispensary	Physician	NPC	Nurse-midwife	Auxiliary staff^a^
Namibia	96.7	96.7	96.7	96.6	97.5	96.6	85.2	100	97.1	–
Kenya	86.5	86.8	85.8	87.7	84.0	86.1	95.5	87.2	86.3	60.7
Tanzania	73.3	73.5	72.8	70.0	68.1	75.6	64.3	75.2	70.2	80.0
Uganda	89.8	90.8	87.8	91.9	87.2	86.1	97.6	91.2	86.4	83.3
Pneumonia	Overall	≤ 24 months	> 24 months	Hospital	Health Centre	Dispensary	Physician	NPC	Nurse-midwife	Auxiliary staff
Namibia	42.5	44.1	40.1	29.9	40.8	43.8	46.3	25.0	42.5	–
Kenya	24.0	25.0	22.2	23.9	27.3	21.9	43.8	26.4	20.2	1.8
Tanzania	24.6	25.8	22.1	29.9	27.7	21.4	22.3	28.5	14.4	20.0
Uganda	19.5	20.4	17.6	20.7	20.0	19.5	19.5	21.9	15.4	0.0
Diarrhoea	Overall	≤ 24 months	> 24 months	Hospital	Health Centre	Dispensary	Physician	NPC	Nurse-midwife	Auxiliary staff
Namibia	29.4	36.0	19.1	32.2	28.9	29.3	40.7	37.5	28.9	–
Kenya	19.4	21.7	15.2	20.4	21.0	16.7	32.6	20.6	17.5	3.6
Tanzania	20.3	23.4	14.0	24.3	20.9	18.2	10.3	23.7	13.6	0.0
Uganda	39.1	42.7	31.3	47.1	32.4	19.5	45.0	46.4	25.7	16.7

*IMCI* Integrated management of childhood illness, *NPC* non-physician clinician

^a^this category did not exist in Namibia

**Table 6:** Adherence (percentage) of health workers to clinical assessment in pneumonia and diarrhoea

	Counting respiration rate/auscultation in all children	Counting respiration rate/ auscultation only in pneumonia patients	Dehydration assessment in all children	Dehydration assessment only in diarrhoea patients
	%	% (n)	%	% (n)
Namibia (*n* = 1495)	42.5	92.2 (128)	29.4	61.5 (325)
Kenya (*n* = 1890)	24.0	87.8 (254)	19.4	41.9 (277)
Tanzania (*n* = 2469)	24.6	82.1 (446)	20.3	42.2 (346)
Uganda (*n* = 1002)	19.5	80.6 (124)	39.1	62.1 (201)

Assessment rates of the IMCI danger signs and main symptoms tended to be higher for children younger than 24 months, at hospitals and when performed by higher-qualified staff (physician/NPC) (Table 4). However, this was not a consistent finding in all countries. Interestingly, NPCs performed better than physicians in many of the assessments. Physical examination rates of diarrhoea exhibited a similar pattern, whereas the examination of pneumonia and fever was better when performed by higher-qualified staff irrespective of the child’s age (Table 5).

In general, health service providers with recent re-training in IMCI (mostly past 12 months; in some categories also past 13 to 36 months) performed better than those without re-training or unknown re-training status. This was a consistent finding in all countries (Table 7). Marked differences were observed for the combined IMCI danger signs and pneumonia assessment. But even then adherence rates were often very low (especially for danger signs, diarrhoea assessment), leaving considerable room for improvement also among health workers with re-training.

**Table 7:** Adherence (percentage) of staff to the IMCI assessment algorithm in relation to IMCI re-training status

	Namibia	Kenya	Tanzania	Uganda
Number of staff with known IMCI training status (n)	185	586	267	204
Number of staff with unknown IMCI training status (n)	255	29	345	135
Children assessed by staff with known IMCI training status (n)	621	1809	1124	635
Children assessed by staff with unknown IMCI training status (n)	878	81	1345	367
IMCI danger signs combined				
IMCI training last 12 months (%)	24.8	21.1	25.1	32.1
IMCI training last 13–36 months (%)	14.6	12.3	19.8	32.1
No IMCI training (%)	8.4	8.4	10.3	24.1
IMCI training status unknown (%)	8.5	2.5	8.8	33.8
IMCI main symptoms combined				
IMCI training last 12 months (%)	55.4	42.5	55.6	49.7
IMCI training last 13–36 months (%)	53.5	33.9	58.8	53.4
No IMCI training (%)	42.5	32.3	41.5	57.5
IMCI training status unknown (%)	47.4	29.6	46.5	62.4
Fever				
IMCI training last 12 months (%)	97.5	89.4	76.2	92.7
IMCI training last 13–36 months (%)	97.2	86.1	74.6	86.9
No IMCI training (%)	96.3	86.2	71.0	90.9
IMCI training status unknown (%)	96.7	81.4	72.5	89.9
Pneumonia				
IMCI training last 12 months (%)	61.1	36.7	42.6	33.2
IMCI training last 13–36 months (%)	66.7	28.5	31.9	16.0
No IMCI training (%)	38.1	20.9	18.4	13.8
IMCI training status unknown (%)	36.8	11.1	18.3	17.4
Diarrhoea				
IMCI training last 12 months (%)	33.9	23.4	25.5	40.2
IMCI training last 13–36 months (%)	26.4	24.5	23.1	44.4
No IMCI training (%)	28.1	17.6	19.1	34.1
IMCI training status unknown (%)	29.5	13.6	18.2	37.0

*IMCI* Integrated management of childhood illness

In all countries, less than 5% of children received a complete/near-complete assessment (11 or 10 items). Health workers in Uganda performed best (median 7 items per child (IQR 5–8)), with Namibia being second (median 6 items per child (IQR 4–7)), whereas in Kenya and Tanzania health workers achieved a median assessment rate of 5 items (IQR 4–7 and 3–7, respectively) per child only. In 55 children, not a single assessment item was performed. In Kenya, physicians performed more items during their assessment than the other health worker groups, whereas in Uganda physicians and NPCs performed better. In Namibia and Tanzania no differences between the health worker cadres were seen.

The median time for conducting the IMCI algorithm was 12 min (IQR 8–28) in Namibia, 9 min (IQR 5–25) in Kenya, 9 min (IQR 5–15) in Tanzania, and 7 min (IQR 5–13) in Uganda. The health workers completed 90% of all assessments within 56 min in Namibia, 80 min in Kenya, 55 min in Tanzania, and 50 min in Uganda.

## Discussion

To the best of our knowledge, our study provides the first analysis of *national* adherence rates to IMCI protocols in LMICs. Despite nationwide training in and expansion of IMCI to the majority of or all districts in the four countries, the adherence rates for assessment of danger signs, main symptoms and for physical examination remained low. The analysis revealed large differences between the countries, with Uganda performing best in the assessment of danger signs, main symptoms and the examination for diarrhoea. Namibia fared best in the examination of fever and pneumonia. Differences in IMCI coverage or time since introduction cannot explain these inter-country differences fully as these factors were comparable across the countries.

Especially when looking at the combined assessment rates of IMCI danger signs and main symptoms, which form the basis for the next steps in the algorithm, health staff did not perform well. In some cases, adherence was better in children ≤ 24 months, at hospitals and health centres, and when performed by higher-qualified staff (physicians/NPCs), but results varied considerably between and within countries. Recent (< 12 months) re-training in IMCI seemed to have a positive effect on adherence rates, but there was still ample room for improvement.

In comparison to our results, studies in IMCI intervention areas, especially from the early period, yielded considerably better results, sometimes reporting adherence rates of more than 90% [10–16, 18]. Typically, these were controlled studies with large numbers of patients and health care providers, performed at a sub-national level (region or district), often with staff specifically trained in IMCI and regularly supervised. The Tanzanian studies showed adherence rates of 95%, especially in the domains of checking for danger signs and physical examination [13, 14]. The studies from Bangladesh showed considerably higher assessment rates for danger signs and main symptoms (> 80%) [10, 11]. Similar results were observed in studies from South Africa, Brazil, Peru and Morocco, but not in Uganda [12, 16–18, 20].

Later on, most studies were performed with varying cadres of health workers and typically fewer patients at sub-national levels (region, district or local community) in sub-Saharan Africa, Asia and South America. Adherence to the different parts of the IMCI algorithm showed a large variance, with percentages ranging from almost zero to close to 100%, with better adherence often during the assessment of danger signs [21–31, 47–50]. Most studies did not find a significant difference in performance between health workers who were trained in long- (11 days) or short-lasting (5–7 days) IMCI courses [50–54]. Some authors noted a decline in performance and adherence rates depending on the time since the last IMCI (re-) training [27, 47, 55], whereas others could not confirm these results [53, 56]. Although our data were collected on a cross-sectional basis, they still indicate that with less frequent or no IMCI re-training health workers showed reduced adherence rates. However, lack of any pre-service or in-service training was consistently identified as a risk factor for poor adherence [10, 11, 13–16].

Only a few studies examined the effect of health workers’ medical qualifications on adherence rates. In our study, physicians and/or NPCs performed better. In other studies, very often NPCs and other health cadres (e.g. nurses, midwives, auxiliary staff) were more frequently trained in IMCI and performed better than physicians [31, 51, 57]. This is an important finding as these cadres conduct the majority of patient assessments, especially in lower-level health institutions and rural areas.

Contrary to the intention of the IMCI system and not reported hitherto, outpatient departments at hospitals performed better than health centres and dispensaries which are both the intended main target institutions for IMCI implementation. This implies that more efforts have to be directed towards improving the quality of care at these lower-level institutions.

Differing adherence rates according to the age of the child, mainly in favour of the children ≤ 24 months (especially for the assessment of the danger sign of ‘inability to drink’ and main symptom of ‘diarrhoea’), have not been reported yet. As an indirect hint towards age preferences, one other study from Tanzania demonstrated that more time was spent on the assessment of children less than 5 years compared to older children [58]. One may speculate that health workers feel more responsible for younger than for older children or that they assume that the younger age group may be more vulnerable and thus deserving of closer assessment, but this assumption would have to be proven in a qualitative prospective study.

In addition, studies from Brazil [59] and other countries [29, 58] have revealed that frequently health workers did not have the time to perform complete assessments due to the high numbers of patients and the overall work load. In our study, health workers used a median duration of 7–12 min per patient which is comparable to the literature [29, 58, 59]. However, at least 10% of the examinations lasted more than 50 min during the surveys which would be far too long for a routine assessment, especially in situations of high patient numbers. Looking at these data, it seems reasonable to assume that the complex IMCI algorithm needs at least the same amount of time as other assessment approaches, but in some instances may require too many resources. This could explain why health workers in this study did not perform all assessment items in the majority of patients [59].

Due to the nature of the survey data, this analysis was only able to provide quantitative data on adherence rates in the four countries. Lange et al. studied qualitative reasons for poor adherence to the IMCI protocols in Tanzania [35]. They found that health workers were often not convinced that IMCI in itself was a valuable approach, sometimes they had a low intrinsic motivation to follow the protocols or lacked the capacity to apply their knowledge to the respective patient (“know-do gap”), or resorted to simple approaches as a rule of thumb. Lack of re-training, poor remuneration, and the overall poor state of the health system seemed to play a role too, which was also reported in other studies [21, 22, 32].

In light of our findings and the literature, poor/low adherence may be one important reason for not achieving the goal of substantially lowering child mortality in LMICs after introduction of IMCI. A recent meta-analysis, based on studies from Tanzania, India and Bangladesh, revealed that instead of an anticipated 50% reduction of under-five mortality, IMCI may have reduced mortality by only 15% [11, 14, 60–62]. Only one study from Egypt found a marked acceleration in the annual reduction of under-five mortality after the introduction of IMCI into the national health system [63]. Other factors include the lack of sufficient numbers of health workers, lack of IMCI-trained staff, high staff turnover, lack of political support and sufficient investment in the health system, and poor implementation of the community component of IMCI in most LMICs [5, 7, 18, 34, 64–66].

### Strengths and limitations

The strengths of the surveys and this analysis include the *representative* national samples from four countries with large numbers of participating health institutions, health workers and patients. The surveys utilised a standardised data collection tool which was comparable across time periods and countries and aligned with the IMCI algorithm, and which covered all levels of the health system and all qualification levels of health workers. Thus the results can be considered to be more representative than other studies which were conducted on a smaller and more selective scale. Furthermore, we believe that these results are closer to the reality than data from intervention studies where special emphasis was placed on IMCI training, performance and supervision.

The limitations of the surveys were the non-randomised approach to the selection of the health workers and the patients and the lack of assessment of the appropriateness of diagnosis and treatment. Still the large numbers of participants and health workers should minimise these limitations. Furthermore, in some countries the data were collected several years ago. Typically it takes a long time (and human and financial resources) to prepare these surveys, to collect the data and to analyse them. Thus, prolonged periods elapse between the surveys in most of the countries. Finally, direct observation may have contributed to increased rates of adherence, but if so, then true adherence rates would be even lower than reported here. Despite these constraints, this analysis provides important insights into the patterns of adherence to IMCI protocols across several sub-Saharan African countries.

## Conclusion

Despite nationwide training in IMCI the adherence rates for assessment and physical examination remained low in all four countries, especially among nurses, midwives and auxiliary staff. The results suggest that adherence rates are particularly low for children older than 24 months and those in non-hospital settings. Also, recent IMCI re-training appears to lead to better adherence rates. In the light of these findings, special attention needs to be directed towards IMCI training of all health staff, with particular emphasis on nurses, midwives, and auxiliary staff, and which is consolidated with periodic re-training. Children of all age groups should be equally targeted for assessment. Our findings call for continuing and increased efforts to improve the standard of paediatric care within the framework of IMCI in LMICs.

## Abbreviations

IMCI: Integrated Management of Childhood Illness
IQR: Interquartile range
KE: Kenya
LMIC: Low-and-Middle-Income Country
NA: Namibia
NPC: Non-physician clinician
SPA: Service provision assessment
TZ: Tanzania
UG: Uganda
USD: US dollars
